# Systematic Identification of Placental Epigenetic Signatures for the Noninvasive Prenatal Detection of Edwards Syndrome

**DOI:** 10.1371/journal.pone.0015069

**Published:** 2010-11-30

**Authors:** Dana W. Y. Tsui, Y. M. Doris Lam, Wing S. Lee, Tak Y. Leung, Tze K. Lau, Elizabeth T. Lau, Mary H. Y. Tang, Ranjit Akolekar, Kypros H. Nicolaides, Rossa W. K. Chiu, Y. M. Dennis Lo, Stephen S. C. Chim

**Affiliations:** 1 The Centre for Research into Circulating Fetal Nucleic Acids, Li Ka Shing Institute of Health Sciences, The Chinese University of Hong Kong, Hong Kong Special Administrative Region, China; 2 Department of Chemical Pathology, The Chinese University of Hong Kong, Hong Kong Special Administrative Region, China; 3 Department of Obstetrics and Gynaecology, The Chinese University of Hong Kong, Hong Kong Special Administrative Region, China; 4 Prenatal Diagnostic and Counselling Department, Tsan Yuk Hospital, Hong Kong Special Administrative Region, China; 5 Harris Birthright Research Centre for Fetal Medicine, King's College Hospital, London, United Kingdom; Stanford University School of Medicine, United States of America

## Abstract

**Background:**

Noninvasive prenatal diagnosis of fetal aneuploidy by maternal plasma analysis is challenging owing to the low fractional and absolute concentrations of fetal DNA in maternal plasma. Previously, we demonstrated for the first time that fetal DNA in maternal plasma could be specifically targeted by epigenetic (DNA methylation) signatures in the placenta. By comparing one such methylated fetal epigenetic marker located on chromosome 21 with another fetal genetic marker located on a reference chromosome in maternal plasma, we could infer the relative dosage of fetal chromosome 21 and noninvasively detect fetal trisomy 21. Here we apply this epigenetic-genetic (EGG) chromosome dosage approach to detect Edwards syndrome (trisomy 18) in the fetus noninvasively.

**Principal Findings:**

We have systematically identified methylated fetal epigenetic markers on chromosome 18 by methylated DNA immunoprecipitation (MeDIP) and tiling array analysis with confirmation using quantitative DNA methylation assays. Methylated DNA sequences from an intergenic region between the *VAPA* and *APCDD1* genes (the *VAPA-APCDD1* DNA) were detected in pre-delivery, but not post-delivery, maternal plasma samples. The concentrations correlated positively with those of an established fetal genetic marker, *ZFY*, in pre-delivery maternal plasma. The ratios of methylated *VAPA-APCDD1*(chr18) to *ZFY*(chrY) were higher in maternal plasma samples of 9 male trisomy 18 fetuses than those of 27 male euploid fetuses (Mann-Whitney test, P = 0.029). We defined the cutoff value for detecting trisomy 18 fetuses as mean+1.96 SD of the EGG ratios of the euploid cases. Eight of 9 trisomy 18 and 1 of 27 euploid cases showed EGG ratios higher than the cutoff value, giving a sensitivity of 88.9% and a specificity of 96.3%.

**Conclusions:**

Our data have shown that the methylated *VAPA-APCDD1* DNA in maternal plasma is predominantly derived from the fetus. We have demonstrated that this novel fetal epigenetic marker in maternal plasma is useful for the noninvasive detection of fetal trisomy 18.

## Introduction

Fetal chromosomal aneuploidies are the main reasons for pregnant women to seek prenatal diagnosis [1]. Definitive diagnosis of fetal aneuploidy often requires obtaining fetal genetic materials by invasive procedures, which carry a risk of procedure-associated fetal loss [2]. The presence of fetal DNA in maternal plasma has opened up new opportunities for noninvasive prenatal diagnosis [3]. However, the noninvasive detection of fetal aneuploidy in maternal plasma is complicated by the low fractional concentration (3–10%) of fetal DNA, which co-exists with a large background of maternal DNA [4], [5].

To detect fetal Down syndrome noninvasively, we and other researchers have applied massively parallel genomic sequencing to determine the proportional amounts of chromosome 21 DNA molecules in maternal plasma [6]–[8]. This approach entails the analysis of millions of DNA molecules, derived from both the mother and the fetus, in maternal plasma. As an alternative to the still technically and bioinformatically complex massively parallel sequencing-based approach, we have developed other approaches by targeting fetal-specific DNA or RNA molecules in maternal plasma [9]–[12].

Previous findings suggest that the placenta is the major source of cell-free fetal nucleic acids in maternal plasma [13]–[15], whereas maternal blood cells are the major source of maternal nucleic acids in maternal plasma [16]. Thus, we have used epigenetic signatures specific to the placenta but not maternal blood cells to target fetal DNA in maternal plasma [14], [17]–[19]. We have developed a placental epigenetic signature, namely the unmethylated promoter of the *serpin peptidase inhibitor*, *clade B (ovalbumin)*, *member 5* (*SERPINB5*, NM_002639) gene, into a fetal epigenetic (DNA methylation) marker. Since this fetal epigenetic marker is located on chromosome 18, it is feasible to detect fetal trisomy 18 by assessing the ratio of the alleles in fetuses who are heterozygous for this marker [20]. However, this approach is only applicable to fetuses polymorphic for the marker. Thus, we developed an alternative approach, the epigenetic-genetic (EGG) chromosome dosage approach, with potentially wider population coverage.

We have recently applied the EGG approach for the noninvasive detection of fetal trisomy 21 [11]. This EGG analysis involves a fetal epigenetic marker, *holocarboxylase synthetase* (*HLCS*, NM_002639) gene, which is found to be hypermethylated in the placenta in comparison to maternal blood cells. Comparing this fetal epigenetic marker, which is located on the chromosome 21, with a fetal genetic marker on a reference chromosome unaffected by trisomy 21, we could infer the relative dosage of fetal chromosome 21 by analyzing maternal plasma DNA [11]. Since any paternally-inherited fetal sequences that are not found in the pregnant woman (e.g. Y-chromosomal sequences for male fetuses or polymorphic sequences inherited only from the father for both male and female fetuses) and are located on a reference chromosome can serve as the fetal genetic marker for the EGG analysis, potentially all fetuses in the general population may be covered.

Yet, if we use the EGG approach to detect the small increase (∼1.5-fold) in the dosage of fetal chromosome 21 relative to any other unaffected fetal chromosome in maternal plasma samples collected from pregnancies involving a Down syndrome fetus, an analytical platform of high precision is required. The bisulfite-based detection method of fetal epigenetic markers can only offer limited precision, because bisulfite has been reported to degrade >90% of the input DNA [21]. A bisulfite-independent method using methylation-sensitive restriction enzyme that digests only unmethylated, but not methylated DNA has been developed to detect methylated fetal epigenetic marker [17]. Adopting this method to detect the methylated *HLCS* DNA molecules in maternal plasma, we have achieved an analytical precision that was high enough to distinguish between trisomy 21 and euploid fetuses noninvasively [11].

Therefore, the fetal epigenetic marker suitable for the EGG approach has to be resistant to digestion by methylation-sensitive restriction enzymes in maternal plasma. In other words, we need a fetal epigenetic marker that is hypermethylated in the placenta, the predominant source of fetal DNA in maternal plasma [14], and is located on the potentially aneuploid chromosome. Most of the earlier studies searched for fetal epigenetic markers only within selected genomic loci on chromosome 21 [11], [22], [23]. Using methylated DNA immunoprecipitation (MeDIP) and tiling array analysis [24], other investigators have expanded the search to chromosome 18 and reported numerous loci as potential fetal epigenetic markers [25]. However, no studies have validated whether these MeDIP-identified markers are detectable in maternal plasma, let alone their fetal-specificity in maternal plasma and clinical application.

In this study, we aimed to identify fetal methylated markers on chromosome 18 by MeDIP in a systematic way, and to confirm its detectability and fetal-specificity in maternal plasma. Furthermore, we applied this marker to develop an EGG test for the noninvasive detection of fetal trisomy 18.

## Materials and Methods

### Objectives

To systematically test if there are any genomic loci on chromosome 18 that are hypermethylated in the placenta, compared with maternal blood cells, we profiled the DNA methylation levels in the two tissue types by MeDIP and tiling array (MeDIP-chip). To test if the methylated DNA of such a locus is fetal specific in maternal plasma, we measured its plasma concentration before and after delivery of the fetus. To test if this methylated fetal epigenetic marker in maternal plasma can be used to detect fetuses with trisomy 18 using the EGG approach, we measured its concentrations relative to a fetal genetic marker by digital PCR.

### Ethics approval

This study was conducted according to the principles expressed in the Declaration of Helsinki. Ethics approval from the Joint Chinese University of Hong Kong-New Territories East Cluster Clinical Research Ethics Committee and the respective institutional review broads was obtained. All patients provided written informed consent for the collection of samples and subsequent analysis.

### Subjects and sample collection

Women with singleton pregnancies attending the respective hospitals in Hong Kong and the UK were recruited (File S1). Placental tissues were collected from first- and third-trimester pregnant women undergoing pregnancy termination and elective cesarean section, respectively. Maternal peripheral blood samples were collected just before and 24 hours after the obstetrics procedures. Additionally, blood samples were collected from first- and second-trimester pregnant women attending antenatal care. The trisomy 18 status of the fetus was confirmed by full karyotyping of chorionic villus samples. Blood samples were also collected from non-pregnant individuals as negative controls for validating the fetal-specificity of the candidate marker in plasma.

### Sample processing

Maternal plasma was harvested from EDTA-blood by our previously established double-centrifugation protocol [26]. The maternal blood cell sample portion was recentrifuged at 2,500×*g* for removal of any residual plasma. Placenta was rinsed thoroughly in phosphate buffered saline to remove blood. DNA was extracted from plasma, blood cells and the placenta with the methods described in File S1.

### MeDIP and tiling array (MeDIP-chip) analysis

The DNA sample was sonicated and subjected to MeDIP by antibody specific for methylated cytosine [24]. The immunoprecipitated product was amplified, labeled and hybridized on the GeneChip Human Tiling 2.0R Arrays (Affymetrix). Genomic loci with significantly higher DNA methylation in the placenta, relative to maternal blood cells, were identified by the Tiling Array Software (TAS) version 1.1 and the Model-based Analysis of Tiling array (MAT) method with the parameters described in File S1 [27], [28].

### Rapid and quantitative DNA methylation analysis by the Epityper

MeDIP-identified locus was analyzed by a quantitative DNA methylation assay, the Epityper (Sequenom) [29](File S1). Briefly, the genomic locus of interest in a bisulfite-converted DNA sample was subjected to PCR amplification by primers listed in Table S1, *in vitro* transcription into RNA, and uracil-specific cleavage on the complementary strand. The product derived from PCR amplicons would therefore be fragmented. Fragments derived from the methylated and unmethylated DNA molecules would have different masses due to the difference in nucleotide sequence at the CpG site caused by bisulfite conversion. The mass differences were readily resolved and quantified as distinct peaks by matrix-assisted laser desorption and ionization time-of-flight (MALDI-TOF) mass spectrometry. In the Epityper, the DNA methylation level of one or more CpG sites in any one cleaved fragment was reported as an integral unit, namely a CpG unit. For each CpG unit, a methylation index (MI) was calculated as the ratio of the methylated peak height to the sum of the methylated and unmethylated peak heights.

Priority for Epityper analysis was given for MeDIP-identified loci that would (i) allow appropriate PCR design, including a high annealing temperature of the primers; (ii) result in Epityper assays with the maximum number of detectable fragments (CpG units), the masses of which were within the detection range of the mass spectrometer; and (iii) allow specific PCR amplification from bisulfite-converted DNA. (i)-(ii) were mainly calculated by the Epidesigner 2.0 program (Sequenom); and (iii) was checked by the BiSearch search tool [30]. Occurrence of genomic variations in the analyzed regions were checked using the Database of Genomic Variants (http://projects.tcag.ca/variation/) [31]. We made sure that there was no reported variant of higher than 1% frequency in the regions we analyzed.

### Single-base single-molecule DNA methylation analysis by bisulfite genomic sequencing

To quantify DNA methylation at the resolution of single CpG site in a single molecule, bisulfite genomic sequencing was performed [32]. Briefly, a genomic locus in the bisulfite-converted DNA was amplified by PCR primers (Table S1). The PCR products were cloned and segregated as colonies, each representing a single molecule for sequencing (File S1). For each CpG site in a DNA sample, a MI was calculated as the ratio of the number of methylated clones to the total number of clones sequenced.

### Conventional quantitative PCR assays for VAPA-APCDD1, ZFY and β-actin DNA

DNA samples, which had been subjected to digestion by methylation-sensitive restriction enzyme (File S1) or mock digestion by 50% glycerol instead of an enzyme, were then analyzed by 3 quantitative polymerase chain reaction (qPCR) assays. One qPCR assay was designed to target an intergenic region between the *VAMP (vesicle-associated membrane protein)-associated protein A*, *33 kDa* (*VAPA*, NM_003574) gene and the *adenomatosis polyposis coli down-regulated 1* (*APCDD1*, NM_153000) gene. Another assay, targeting the *zinc finger protein*, *Y-linked* (*ZFY*, NM_003411), an established genetic marker for detecting fetal DNA in maternal plasma of pregnancies bearing male fetuses, was adopted from our previous study [5]. The third assay was designed to target a completely unmethylated region (positive control for methylation-sensitive restriction enzyme digestion) of the *β-actin* (*ACTB*, NM_001101) gene.

All 3 assays involved hydrolysis probes for detection. The primer and probe sequences and reaction conditions are listed in Tables S2 and S3. Concentrations of each target were quantified by a standard calibration curve constructed with known concentrations of a male genomic DNA sample. Any signals detected below the limit of detection (3 copies/PCR for all 3 qPCR assays), as determined by the method in File S1, were considered undetectable. Eight no template controls (water only) were included in every PCR run.

### Digital PCR assays for EGG dosage analysis

Since high analytical precision is required for chromosome dosage analysis, this part of the study was performed by digital PCR [33]. We performed digital PCR through the dilution of the DNA sample to an average concentration of one template molecule in every two reaction wells [34]. This diluted DNA sample was then distributed to hundreds of reaction wells on a 384-well plate for PCR amplification of *VAPA-APCDD1*, *ZFY* and *β-actin* DNA. Primer and probe sequences and reaction conditions are listed in Tables S2 and S3. The actual number of template molecules was calculated by the direct counting of the number of positive wells followed by correction for the Poisson distribution (File S1).

### Statistical analysis

Statistical analyses were performed with the Sigma Stat v3.5 (Systat).

## Results

### Systematic identification of chromosome 18 loci hypermethylated in the placenta by MeDIP and the Epityper

We have embarked on studying DNA methylation levels of the entire chromosome 18, which contains about 351,500 CpG sites in 74.7 million bases of non-repetitive DNA sequence [35]. Ten DNA samples from 5 first-trimester euploid placentas and 5 first-trimester maternal blood cell samples were subjected to MeDIP and tiling array analysis. The array interrogated DNA methylation level every 35 bases (average inter-probe distance) on essentially all the non-repetitive DNA sequences of the entire chromosome.

Genomic loci with a signal log ratio between the placenta and maternal blood cells of >0.4 in the TAS analysis or with a P<10^−5^ in the MAT analysis were considered as hypermethylated in the placenta. We identified 3,043 CpG sites located in 178 loci (68 and 110 loci from the TAS and MAT algorithms, respectively) with higher DNA methylation levels in the placenta, relative to maternal blood cells. We found that 140 loci (79%) were located within genes and the remaining 38 loci (21%) were located in the intergenic regions. The results from the TAS and MAT algorithms are listed in Table S4 and Table S5, respectively. The data discussed in this publication have been deposited in NCBI's Gene Expression Omnibus [36], and are accessible through GEO Series accession number GSE22837.

#### Selecting for CpG sites with high DNA methylation levels in the placenta

**Figure 1:**
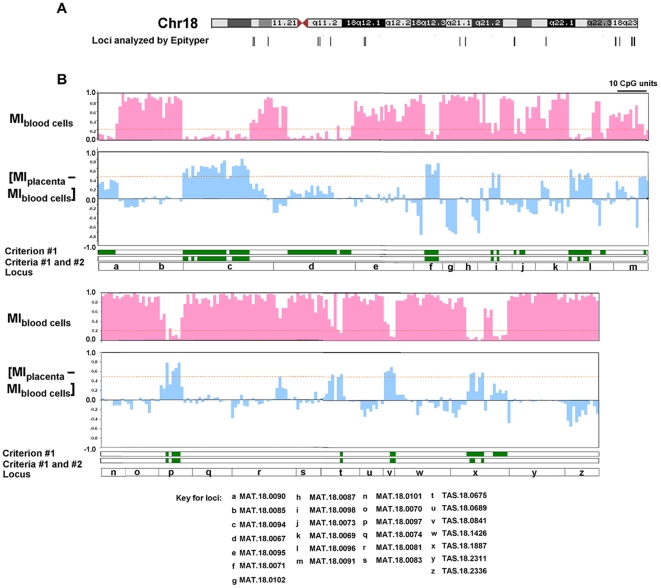
Quantitative DNA methylation levels by the Epityper. (A) Genomic locations of the loci (vertical bars) that were analyzed by the Epityper. (B) Two tracks, each with 5 panels, are shown. *First panel (from top)*. Bar graph of methylation indices (MI) in maternal blood cells (Y-axis) against the chromosomal locations of the CpG units (X-axis, a categorical axis) within the indicated locus (a-z, fifth panel). Each bar represents one CpG unit. The chromosomal locations and MI of each CpG unit are listed in Table S6. A CpG unit is potentially useful for marker development if it fulfills criterion #1: MI in maternal blood cells ≤0.2 (lower than the dotted line, highlighted as green bars in the *third panel*). *Second panel*. Bar graph of the differences in MI between the placenta and maternal blood cells (Y-axis) against the chromosomal locations of the CpG units (X-axis). A CpG unit is most suitable for marker development if it further fulfills criterion #2: MI difference ≥0.5 (higher than the dotted line, highlighted as green bars in the *fourth panel*).

Quantitative DNA methylation levels at the resolution of CpG sites are required for developing fetal epigenetic markers in maternal plasma. For this purpose, we analyzed the MeDIP-identified loci by quantitative DNA methylation assays, the Epityper, which were less labor-intensive and time-consuming than bisulfite sequencing, because it did not involve cloning and sequencing [29], [37]. Priority was given for 370 CpG units (located in 26 loci) which were most efficiently analyzed by the Epityper platform (Figure 1A). For each CpG unit, we compared the DNA methylation levels of two first-trimester placentas, with two first-trimester maternal blood cell samples (Figure 1B and Table S6).

**Figure 2:**
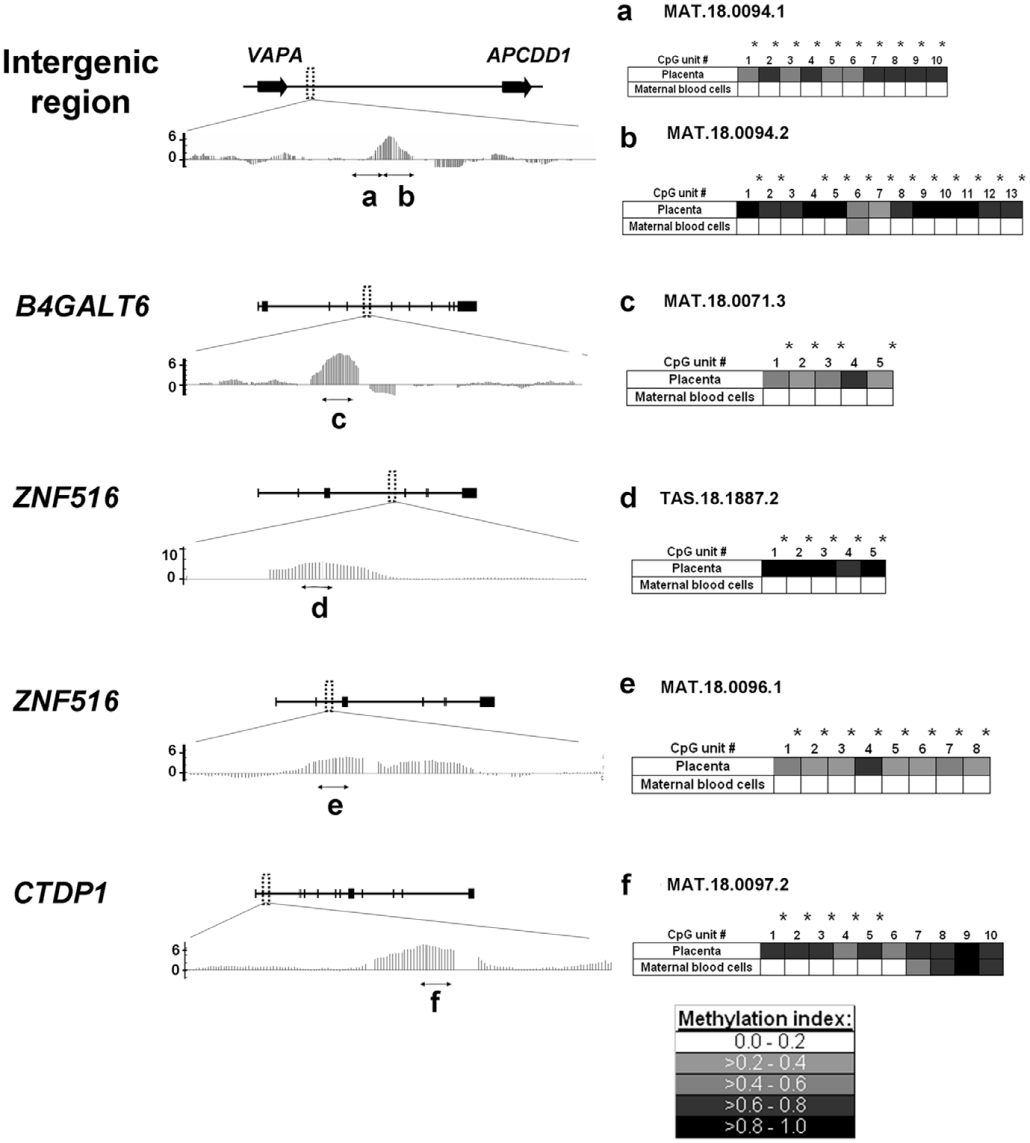
DNA methylation levels by MeDIP and the Epityper on 5 promising loci for marker development. *Left panels*. The positions of these 5 loci (dotted-line boxes) in relation to the associated genes, and their exons (blocks) and introns (lines) are shown. The genes are shown in the direction of mRNA transcription from left to right. The bar graph below each gene shows the difference in the MeDIP-chip probe signals between the placenta and maternal blood cells plotted against chromosomal location. Each vertical bar represents the signal from one probe. The average inter-probe distance is 35 bp. A positive value implies a higher DNA methylation level in the placenta, compared with maternal blood cells. Horizontal lines with arrows at both ends indicate the regions spanned by the Epityper assays (a–f). *Right panels*. The median DNA methylation indices (MI) of CpG units within the Epityper assays are shown in a grey intensity scale. Asterisks indicate the CpG units with higher DNA methylation in five placentas than five maternal blood cell samples (Mann-Whitney test, *P*<0.05). The chromosomal locations and MI of each CpG unit are listed in Table S7.

To select CpG units potentially useful for developing fetal epigenetic markers in maternal plasma, we identified CpG units with a methylation index (MI) ≤0.20 in the maternal blood cells (criterion #1), and with a difference in MI of ≥0.50 between the placenta and maternal blood cells (criterion #2). These criteria were adopted with slight modification from our previous study in developing fetal markers in maternal plasma [22]. Among the 370 CpG units analyzed by the Epityper, 40 CpG units, located in 8 MeDIP-identified loci, fulfilled both criteria #1 and #2 (Table 1; Figure 1B, highlighted as green bars). Three or more potentially useful CpG units were found in each of 5 MeDIP-identified loci (Table 1, top 5 loci). Thus, we further confirmed these observations in more cases. The majority of the analyzed CpG units in these 5 loci were confirmed to possess significantly higher DNA methylation levels in 5 first-trimester placentas, relative to 5 first-trimester maternal blood cell samples (Figure 2; Table S7, Mann-Whitney test, p<0.05).

**Table 1:** MeDIP-identified loci with CpG sites useful for developing fetal epigenetic markers in maternal plasma.

Locus ID	Number of CpG fulfilling criteria #1 and #2	Chromosomal location on chr 18	Associated gene symbol	Associated gene	RefSeq accession number, region
MAT.18.0094	19	10022563-10023955	*VAPA-APCDD1*	*VAMP (vesicle-associated membrane protein)-associated protein A*, *33kDa*, and *Adenomatosis polyposis coli down-regulated 1*	NM_003574 and NM_153000, intergenic region
MAT.18.0071	5	27485628-27487511	*B4GALT6*	*Beta-1,4-galactosyltransferase 6*	NM_004775, intron
MAT.18.0097	4	75542484-75543900	*CTDP1*	*Carboxy-terminal domain*, *RNA polymerase II*, *polypeptide A phosphatase*, *subunit 1*	NM_004715, intron
MAT.18.0096	4	72292238-72293387	*ZNF516*	*Zinc finger protein 516*	NM_014643, intron
TAS.18.1887	3	72227405-72228513	*ZNF516*	*Zinc finger protein 516*	NM_014643, intron
TAS.18.0841	2	27486040-27487378	*B4GALT6*	*Beta-1,4-galactosyltransferase 6*	NM_004775, intron
MAT.18.0098	2	54075774-54077141	*NEDD4L*	*Neural precursor cell expressed, developmentally down-regulated 4-like*	NC000018.9, intron
TAS.18.0675	1	19035725-19036654	*CABLES1*	*Cdk5 and Abl enzyme substrate 1*	NM_138375, intron

Genomic locations are defined according to the human genome database in the UCSC Genome Browser (March 2006 assembly, hg18). chr, chromosome.

#### Selecting for CpG sites with low inter-individual variation in placental DNA methylation levels

**Figure 3:**
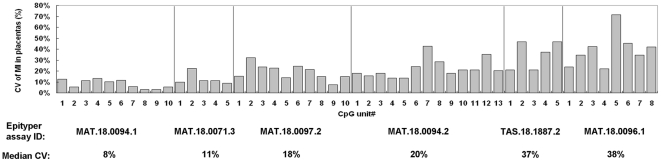
Inter-individual variation of placental methylation levels in promising regions for marker development. For each CpG unit, the coefficients of variation (CV) of the MI in 10 euploid first-trimester placentas are plotted. For each region, the median CV of all CpG units is shown.

We then assessed the inter-individual variations of the MI of the 5 selected loci in 10 first-trimester placental tissue samples. These 5 loci were interrogated by 6 Epityper assays (Figure 3). For each CpG unit, a coefficient of variation (CV * = * standard deviation (SD)/mean) of the MI in 10 placentas was calculated (Figure 3 and Table S8). For each Epityper assay, a median CV of all the CpG units within the assay was also calculated. The 4 Epityper assays with the lowest median CV were selected for further investigation (Figure 3, first 4 assays).

These 4 Epityper assays were performed on 6 euploid and 6 trisomy 18 placental tissue samples. No significant difference between the two groups were found in the methylation of any CpG unit, suggesting that placental DNA methylation was not altered by trisomy 18 (Mann-Whitney test, P>0.05; Table S9).

#### Selecting for CpG sites overlapping with methylation-sensitive restriction enzyme sites

**Figure 4:**
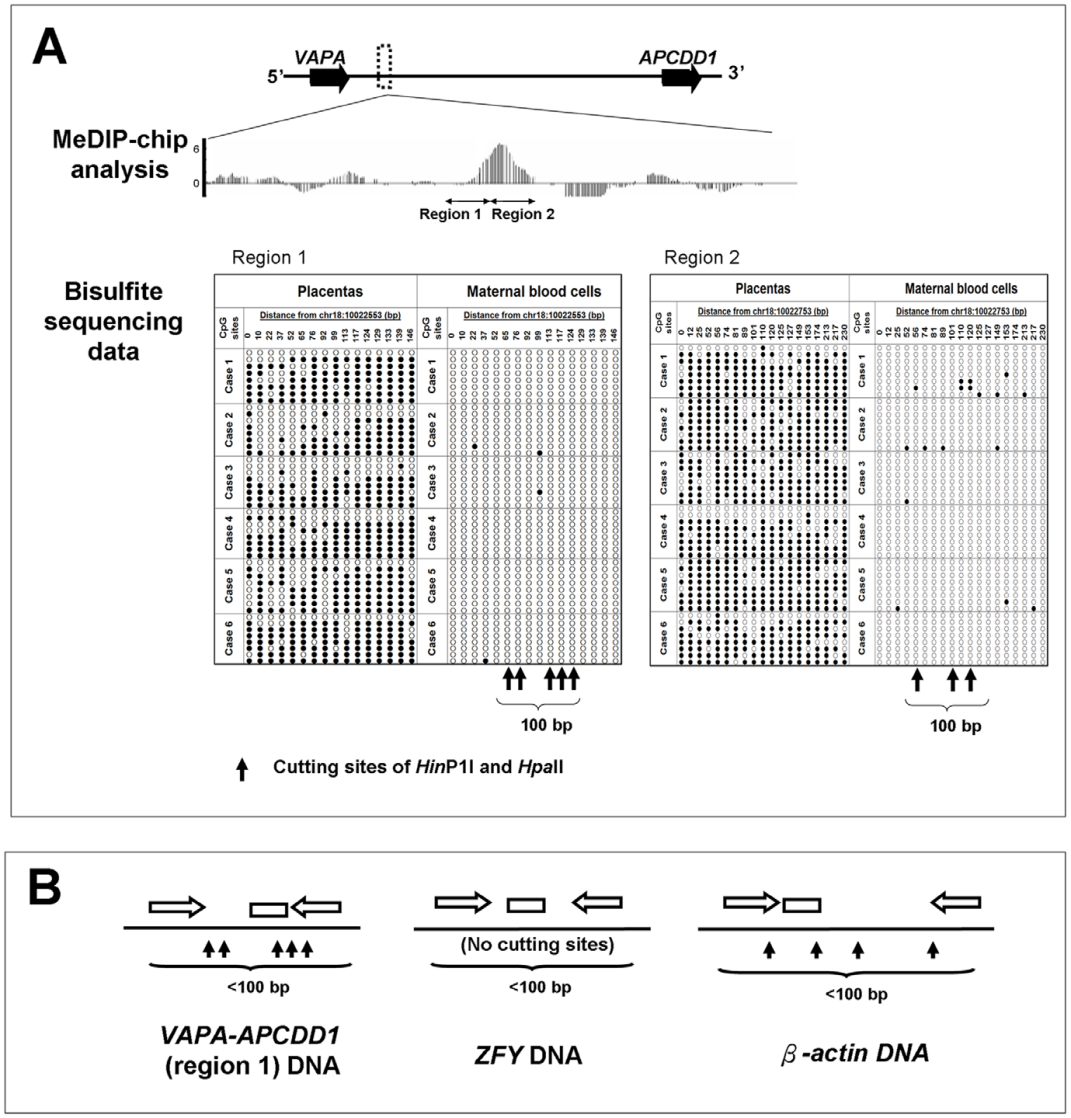
DNA methylation levels by bisulfite sequencing in the most promising locus for marker development. (A) *Top panel*. The genomic location of one promising locus in relation to two associated genes. The locations of two other promising loci are shown in Figure S1. *Middle panel*. Two regions, which were identified by MeDIP as possessing higher methylation in the placenta relative to maternal blood cells, was analyzed by bisulfite sequencing. See Figure 2 for the legend on the bar graph for MeDIP. *Bottom panel*. Single-base DNA methylation levels determined by bisulfite sequencing. For each sample, 8 randomly-picked clones (rows) were scored for each CpG site (column). Filled circles, methylated CpG sites. Empty circles, unmethylated CpG sites. Upward arrows, cutting sites of the methylation-sensitive restriction enzymes *Hpa*II and *Hin*P1I. (B) Design of qPCR and digital PCR assays for region 1 of *VAPA-APCDD1* (5 cutting sites), *ZFY* (0 cutting sites), and *β-actin* DNA (4 cutting sites). Block arrows, PCR primers. Rectangle, hydrolysis probe.

To select specific CpG sites that would be most promising as a noninvasive fetal DNA marker, we performed bisulfite sequencing on the 4 selected regions. Consistent with the MeDIP and the Epityper data, most of the sequenced CpG sites were predominately methylated in the placenta and almost completely unmethylated in maternal blood cells (Figures 4A and S1). We examined these CpG sites for any overlapping sites recognized by two commonly used methylation-sensitive restriction enzymes, *Hin*P1I and *Hpa*II. There were 5, 3, 1 and 1 such sites within 100 bp from the following regions: MAT.18.0094.1 (*VAPA-APCDD1* region 1), MAT.18.0094.2 (*VAPA-APCDD1* region 2), MAT.18.0071.3 (*B4GALT6*), and MAT.18.0097.2 (*CTDP1*), respectively (Figures 4B and S1, upward arrows). Thus, MAT.18.0094.1 was selected for developing a fetal-specific assay in maternal plasma, because the highest number of such sites would facilitate the most complete removal of the unmethylated maternal DNA.

This MeDIP-identified region, MAT.18.0094.1, is an intergenic region located 73 kb downstream of the *VAPA* gene and 421 kb upstream of the *APCDD1* gene (Figure 4A). Hence, we refer to this locus as *VAPA-APCDD1*.

### Detection and characterization of digestion-resistant VAPA-APCDD1 DNA in maternal plasma

We designed a qPCR assay to target region 1 of *VAPA-APCDD1*. Since the PCR primers flanked 5 methylation-sensitive *Hin*P1I and *Hpa*II sites, only *VAPA-APCDD1* molecules methylated in all 5 CpG sites would result in amplifiable qPCR signal (Figure 4B). Thus, the concentration of digestion-resistant *VAPA-APCDD1* DNA detected by this qPCR assay is reflective of the level of the methylated DNA of this potential marker.

#### Concentrations of digestion-resistant *VAPA-APCDD1* DNA in first-, second-, and third-trimester maternal plasma

**Figure 5:**
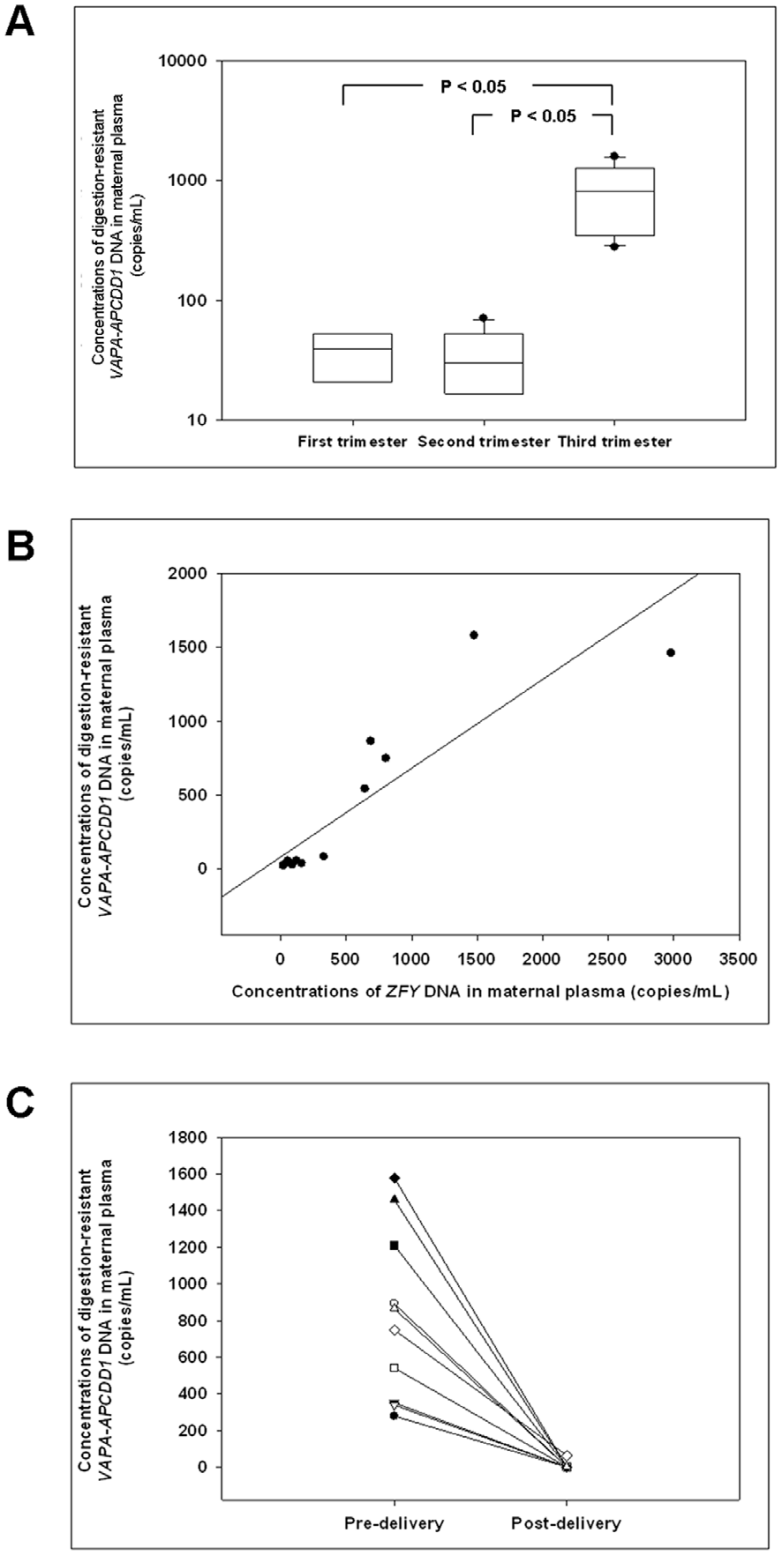
Characteristics of digestion-resistant *VAPA-APCDD1* DNA in maternal plasma. (A) Box plot of the concentrations of this fetal epigenetic marker in the first-, second- and third-trimester pre-delivery maternal plasma. The line inside each box denotes the median. Limits of the box denote the 25^th^ and 75^th^ percentiles. Whiskers denote the 10^th^ and 90^th^ percentiles. Filled circles denote the outliers. The results of the Kruskal-Wallis ANOVA test showed that there are significant differences among the three trimesters (P<0.001). The P-values of the pairwise comparisons with significant differences are shown (Dunn's test). (B) Concentrations of the epigenetic (Y-axis) and genetic (X-axis) markers in 13 maternal plasma samples (Spearman rank order correlation test, P<0.00001, R = 0.91). (C) The concentrations of this marker in 10 pairs of pre-delivery and post-delivery maternal plasma (Wilcoxon Signed-rank test, P = 0.002). Corresponding plasma samples from the same pregnant women are connected by a line.

Using the above qPCR assay, we quantified the *VAPA-APCDD1* DNA in *Hin*P1I and *Hpa*II-digested maternal plasma samples obtained from the three trimesters. Digestion-resistant *VAPA-APCDD1* DNA was readily detected in 26 maternal plasma samples (5/6 (83%) first-trimester, 8/10 (80%) second-trimester and 10/10 (100%) third-trimester samples). The concentrations of the digestion-resistant *VAPA-APCDD1* DNA in the three trimesters were statistically significantly different (Kruskal-Wallis ANOVA test, P<0.001) (Figure 5A).

#### Correlation between digestion-resistant *VAPA-APCDD1* DNA and an established fetal genetic marker in maternal plasma

To determine if there was any correlation between the concentrations of digestion-resistant *VAPA-APCDD1* DNA and those of the *ZFY* DNA, an established genetic marker for detecting male fetal DNA in maternal plasma, we analyzed the maternal plasma samples from 13 pregnant women bearing male fetuses in the previous experiment. Digestion-resistant *VAPA-APCDD1* and *ZFY* concentrations were positively correlated (r = 0.91; P<0.00001; Spearman correlation) (Figure 5B). Both assays were optimized to run under identical PCR thermal profiles (Table S3) and showed similar efficiencies as reflected by the slopes (−3.77 and −3.53 for the *VAPA-APCDD1* and the *ZFY* assays, respectively) and y-intercepts (39.7 Cq and 38.9 Cq for the *VAPA-APCDD1* and the *ZFY* assays, respectively) of the two calibration curves.

#### Postpartum clearance of digestion-resistant *VAPA-APCDD1* DNA in maternal plasma

To further investigate if digestion-resistant *VAPA-APCDD1* DNA in maternal plasma would be cleared upon delivery of the fetus, we collected pre-delivery and 24-hour post-delivery maternal plasma samples from 10 other pregnant women. In all 10 cases, the digestion-resistant *VAPA-APCDD1* DNA was rapidly cleared from maternal plasma to almost undetectable levels (Figure 5C), demonstrating that its existence in maternal plasma was fetal-specific. The plasma concentrations of digestion-resistant *VAPA-APCDD1* DNA before and after delivery of the fetuses were statistically significantly different (Wilcoxon signed-rank test, P = 0.002). As a control for successful DNA extraction, we quantified the total DNA amount by the *VAPA-APCDD1* DNA qPCR assay, and detected DNA in all pre-delivery and post-delivery maternal plasma samples before enzyme digestion. As a control for complete enzyme digestion, a qPCR assay was designed to target a region on the *β-actin* gene, which was known to be completely unmethylated (MI = 0.00) in both the placenta and maternal blood cells (Figures 4B and S2), and no signals were detected in any of the *Hin*P1I- and *Hpa*II-digested maternal plasma samples. The lower limits of detection of the *β-actin* and *VAPA-APCDD1* qPCR assays were both 3 copies per reaction, as determined by the methods in File S1.

### Epigenetic-genetic (EGG) dosage analysis of fetal chromosome 18 in maternal plasma by digestion-resistant VAPA-APCDD1 DNA

#### Digital PCR assays for the *VAPA-APCDD1*, *ZFY* and *β-actin* DNA

To detect the relatively small (∼1.5-fold) increase in the dosage of chromosome 18 in trisomy 18, the digital PCR platform, which featured a higher precision than conventional qPCR, was used. These digital PCR assays were first validated in *Hin*P1I and *Hpa*II-digested plasma samples collected from 5 pregnant women before and after delivery, and 4 non-pregnant females. By these digital PCR assays, digestion-resistant *VAPA-APCDD1* DNA and *ZFY* DNA were detected in pre-delivery maternal plasma samples, but were almost undetectable in post-delivery maternal plasma and non-pregnant female plasma samples (Table S10). *β-actin* DNA was not detected in all 3 groups of digested samples (Table S11).

#### EGG dosage analysis of chromosome 18 in the placenta

Since the placenta is the main source of fetal DNA in maternal plasma, we tested EGG analysis in the placenta before attempting it in maternal plasma. Digital PCR assays for *VAPA-APCDD1* and *ZFY* DNA were performed on *Hin*P1I- and *Hpa*II-digested DNA samples extracted from placental tissues of five trisomy 18 and five euploid male fetuses. The ratio of digestion-resistant *VAPA-APCDD1* to *ZFY* was calculated for each sample. These ratios were significantly higher in the trisomy 18 placentas than the euploid placentas (Mann-Whitney test, P = 0.029; Figure S3). A reference interval, defined as the mean ratio digestion-resistant *VAPA-APCDD1* to *ZFY* ±1.96 SD, was calculated from the euploid placentas as 1.20–1.66. The ratios in all of the trisomy 18 placenta were above the upper reference limit (Figure S3).

#### EGG dosage analysis of fetal chromosome 18 in maternal plasma

**Figure 6:**
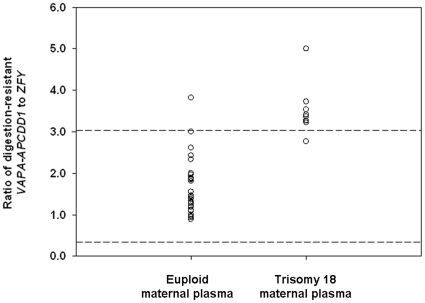
Comparison of chromosome dosage in DNA samples from euploid and trisomy 18 maternal plasma. For each sample, the ratio of digestion resistant *VAPA-APCDD1* DNA (chr18) and *ZFY* DNA (chrY) is plotted. The reference interval of the euploid ratios was calculated as 0.34–3.04 (bound by the dotted lines).

Since the digestion-resistant *VAPA-APCDD1* DNA and *ZFY* DNA are fetal-specific in maternal plasma, we reasoned that EGG dosage analysis of the fetus or the placenta could also be performed in maternal plasma. Maternal plasma samples were collected from 27 women with euploid male fetuses, and 9 women with trisomy 18 male fetuses. The median gestational ages at sample collection were 14.1 weeks (IQR, 12.9–16.6) and 13.3 weeks (IQR 12.9–14.5) among the euploid and trisomy 18 fetuses, respectively. The *Hin*P1I- and *Hpa*II-digested plasma samples were subjected to digital PCR assays for *VAPA-APCDD1* and *ZFY* DNA (Figure S4). To facilitate a fair comparison, we diluted each plasma sample to a comparable average template concentration (*m*) of *ZFY* (reference) molecules per reaction well. The median *m* values were 0.08 and 0.05 per reaction well for the euploid and trisomy samples, respectively. The ratio of digestion-resistant *VAPA-APCDD1* to *ZFY* was calculated for each sample. The ratios of digestion-resistant *VAPA-APCDD1* to *ZFY* were significantly higher in maternal plasma samples of trisomy 18 fetuses than those of euploid fetuses (Mann-Whitney test, P<0.001) (Figure 6). A reference interval of 0.34–3.04 was calculated for the 27 maternal plasma samples from euploid pregnancies. The ratio of one euploid sample fell outside the reference interval. The ratios of 8 out of 9 trisomy 18 samples were above the upper reference limit.

*β-actin* DNA was essentially undetectable by the digital PCR assay in all of the 36 digested maternal plasma DNA samples, implying that complete digestion had occurred. Ten cases were selected for comparing the detectable concentrations of *β-actin* DNA before and after digestion. *β-actin* DNA was detectable in all the maternal plasma samples before, but not after, digestion (Figure S5).

## Discussion

Using MeDIP-chip analysis, the Epityper and bisulfite sequencing, we have systematically identified methylated fetal epigenetic markers on chromosome 18. We have also demonstrated that one such marker, namely the digestion-resistant *VAPA-APCDD1* DNA, was readily detectable in maternal plasma during pregnancy, but rapidly cleared to almost undetectable levels upon delivery of the fetus. Further characterization has shown that the concentrations of this novel marker in maternal plasma were positively correlated with those of an established fetal genetic marker.

Since these data have suggested that this digestion-resistant *VAPA-APCDD1* DNA was predominantly derived from the fetus in maternal plasma, we further applied it for the EGG dosage analysis of fetal chromosome 18 in maternal plasma. We have determined the ratio of digestion-resistant *VAPA-APCDD1* to *ZFY* in maternal plasma samples involving 9 trisomy 18 male fetuses, and 27 euploid male fetuses. A reference interval of this ratio calculated from the euploid samples was calculated. We then observed the ratios of 8 trisomy 18 fetuses, and only 1 euploid fetus was higher than the upper reference limit. Hence using the upper reference limit as a threshold, all except one trisomy 18 fetuses was detected (i.e. 1 false negative), and only one euploid was also detected (i.e. 1 false positive). The sensitivity and specificity were 88.9% and 96.3%, respectively, for this EGG analysis in maternal plasma.

Of note, in this EGG analysis, we have quantified the reference chromosome by a fetal genetic marker, i.e. the *ZFY* DNA, instead of a fetal epigenetic marker, e.g. the hypermethylated *RASSF1A* [17]. This is because we have previously shown that the dosage analysis had greater power in distinguishing the trisomic from the euploid cases, if the reference chromosome was quantified by a fetal genetic marker instead of a fetal epigenetic marker [11]. For fetal epigenetic markers, there are small degrees of heterogeneity in the DNA methylation levels between samples. If we determined the relative ratio between an epigenetic marker on the aneuploid chromosome and another epigenetic marker on a reference chromosome, the combined variance caused by the heterogeneous DNA methylation levels in both epigenetic markers would be large. The resultant inter-individual variation in the chromosome dosage ratio would be too large to discriminate trisomy from euploid cases. Thus, it is preferable to quantify the reference chromosome by a fetal genetic marker.

Of a similar concern, to minimize the inter-individual variability in the DNA methylation level of the fetal epigenetic marker used for quantifying the potentially aneuploid fetal chromosome 18, we have studied how the DNA methylation levels of each CpG unit varied across different individuals by the Epityper. Among the six potential markers, the median of the inter-individual CVs was observed to vary from 8% to 38% (Fig. 3). The data suggested that the development of fetal epigenetic markers by MeDIP-based study should be followed up by quantitative DNA methylation studies at single CpG resolution. Otherwise, a low degree of inter-individual variation, which is important for developing quantitative applications such as the assays for the EGG analysis, could not be guaranteed by the MeDIP-based study alone.

Additionally, our data have suggested that not every CpG site in the MeDIP-identified regions could fulfill the stringent criteria for developing fetal epigenetic marker in maternal plasma. Some CpG sites might possess undesirably high levels of DNA methylation in the maternal blood cells (Figure 1B, not fulfilling criterion #1), thus preventing the removal of the maternal DNA sequence in maternal plasma. On the other hand, some CpG sites might not have high enough DNA methylation levels in the placenta relative to maternal blood cells, thus preventing the specific detection of the fetal DNA sequence in maternal plasma (Figure 1B, not fulfilling criterion #2). Thus, the quantitative DNA methylation data provided by the Epityper for each CpG unit within the MeDIP-identified regions were indispensable for developing fetal epigenetic markers (Figure 3). The Epityper data have facilitated us to select for a suitable epigenetic signature, with marked difference between the placenta and maternal blood cells and with the least inter-individual variation, to target the fetal chromosome 18 in maternal plasma for the EGG analysis.

In this study, we performed the EGG analysis only in pregnancies involving male fetuses because a precise digital PCR assay targeting the Y-chromosome, namely the *ZFY* DNA assay, is well established in our laboratory [5]. However, we envision that this type of EGG analysis can be adopted for pregnancies involving female fetuses, because any fetal DNA sequence, including an autosomal sequence, that is inherited only from the father, and is located on a chromosome unaffected by the concerned trisomy, can serve as a fetal genetic marker for quantifying the reference chromosome. Through the latter approach, EGG analysis can be performed for both female and male fetuses.

In this study, we demonstrated the feasibility of applying the EGG approach for the noninvasive prenatal detection of fetal trisomy 18. The diagnostic accuracy of the test requires further evaluation in a larger cohort. Nevertheless, we are the first to use a bisulfite-independent approach to detect a methylated epigenetic marker for fetal chromosome 18 in first-trimester maternal plasma, and to use this approach to achieve the noninvasive detection of fetal trisomy 18 in early gestation. Compared with tests based on massively parallel genomic sequencing, the approach described here might represent a lower ‘barrier for entry’ by many laboratories interested in noninvasive prenatal diagnosis, as the equipment and bioinformatics support requirements are much lower [38].

## Supporting Information

Figure S1 **DNA methylation levels by bisulfite sequencing in two promising loci for developing fetal epigenetic markers.** Data on each locus are shown in two panels, each of which contains 3 sub-panels. *Top sub-panel*. The genomic location of the promising locus in relation to the associated gene. *Middle sub-panel*. The locus, which was identified by MeDIP as possessing higher methylation in the placenta relative to maternal blood cells, was analyzed by bisulfite sequencing. See Figure 2 for the legend on the bar graph for MeDIP. *Bottom sub-panel*. Single-base DNA methylation levels by bisulfite sequencing. For each sample, 8 randomly-picked clones (rows) were scored for each CpG site (column). Filled circles, methylated CpG sites. Empty circles, unmethylated CpG sites. Upward arrows, cutting sites of the methylation-sensitive restriction enzymes *Hpa*II and *Hin*P1I.
(TIFF)

Figure S2 **DNA methylation levels by bisulfite sequencing in the** ***β-actin*** **gene.** For each sample, 8 randomly-picked clones (rows) were scored for each CpG site (column). Filled circles, methylated CpG sites. Empty circles, unmethylated CpG sites. Upward arrows, cutting sites of the methylation-sensitive restriction enzymes *Hpa*II and *Hin*P1I.
(TIFF)

Figure S3 **Comparison of chromosome dosage in DNA samples from euploid and trisomy 18 placental tissues.** For each sample, the ratio of digestion-resistant *VAPA-APCDD1* DNA (chr18) and *ZFY* DNA (chrY) is plotted. The reference interval of the euploid ratios was calculated as 1.20–1.66 (bound by the dotted lines).
(TIFF)

Figure S4 **Workflow of the EGG chromosome dosage analysis of maternal plasma samples.** Methylation-sensitive restriction enzymes, *Hin*P1I and *Hpa*II. *VAPA-APCDD1/ZFY* assay, a duplex digital PCR assay.
(TIFF)

Figure S5 **Concentrations of** ***β-actin*** **DNA in EGG-analyzed plasma samples before and after enzyme digestion.** *β-actin* DNA was essentially undetectable by the digital PCR assay in any of the 36 EGG-analyzed plasma samples after digestion. Further analysis of ten maternal plasma samples (8 euploid and 2 trisomy 18 cases) before digestion was also performed by this digital PCR assay. Data of these 10 paired samples are shown.
(TIFF)

Table S1 PCR primer sequences of the Epityper assays and bisulfite sequencing.
(XLS)

Table S2 Sequences of PCR primers and hydrolysis probes for qPCR and digital PCR assays.
(XLS)

Table S3 Reaction conditions for qPCR and digital PCR assays.
(XLS)

Table S4 Hypermethylated regions (68 loci) in the placenta identified by the TAS algorithm.
(XLS)

Table S5 Hypermethylated regions (110 loci) in the placenta identified by the MAT algorithm.
(XLS)

Table S6 DNA methylation levels in 370 CpG units by the Epityper.
(XLS)

Table S7 DNA methylation levels of selected loci in 5 placentas and 5 maternal blood cells.
(XLS)

Table S8 DNA methylation levels of selected loci in 10 placentas.
(XLS)

Table S9 DNA methylation levels of selected loci in 6 trisomy 18 and 6 euploid placentas.
(XLS)

Table S10 Digital PCR for fetal epigenetic and genetic markers in 3 types of plasma samples.
(XLS)

Table S11 Digital PCR results for *β-actin* in *Hin*P1I and *Hpa*II-digested plasma samples.
(XLS)

File S1 Supplementary methods.
(DOC)
