# Supplementary figures and images for: Systematic Identification of Placental Epigenetic Signatures for the Noninvasive Prenatal Detection of Edwards Syndrome
Source: PLoS One. 2010 Nov 30;5(11):e15069. doi: 10.1371/journal.pone.0015069 (PMC2994810; doi:10.1371/journal.pone.0015069)

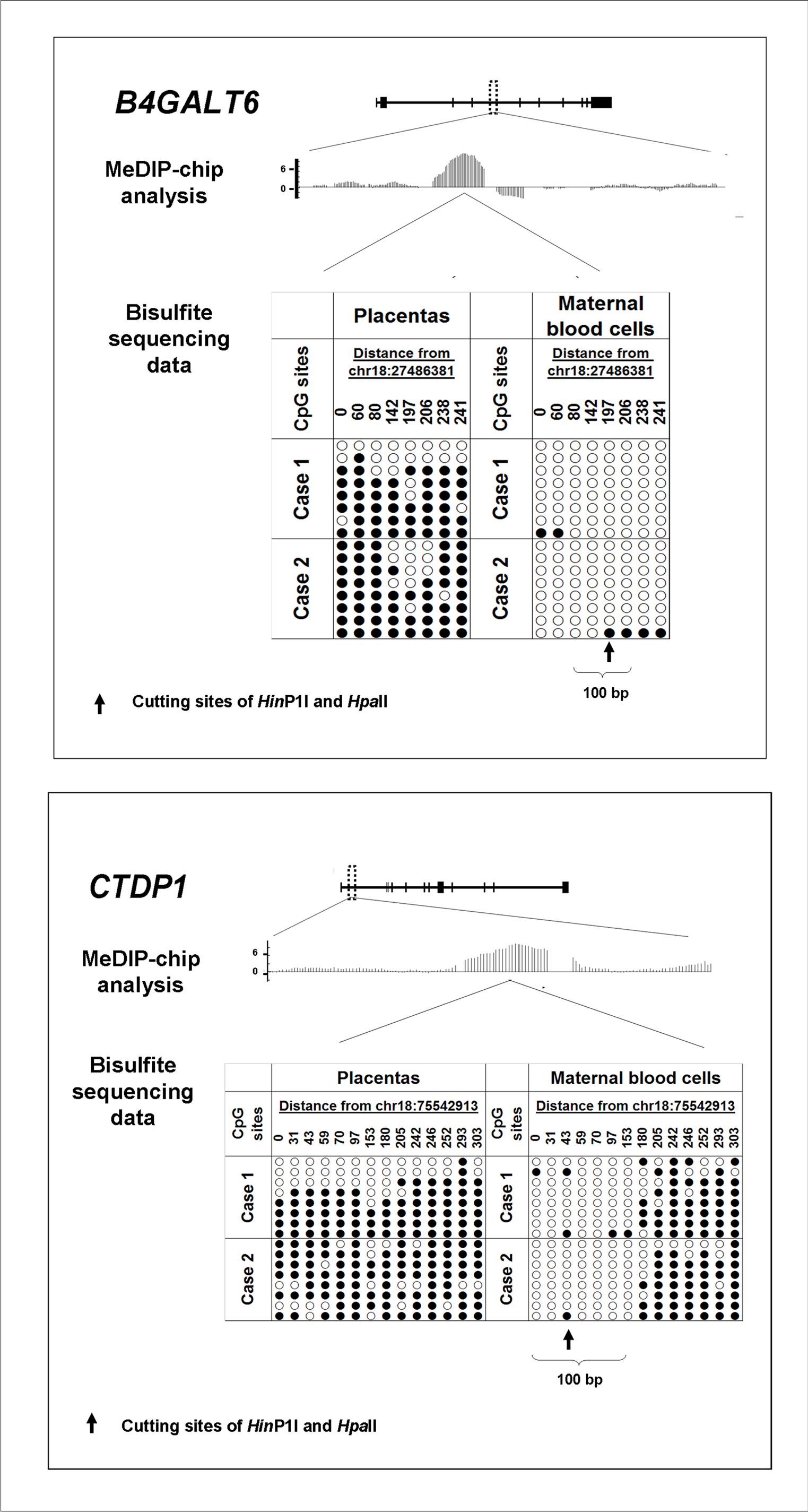

Supplement: Figure S1 — DNA methylation levels by bisulfite sequencing in two promising loci for developing fetal epigenetic markers. Data on each locus are shown in two panels, each of which contains 3 sub-panels. Top sub-panel. The genomic location of the promising locus in relation to the associated gene. Middle sub-panel. The locus, which was identified by MeDIP as possessing higher methylation in the placenta relative to maternal blood cells, was analyzed by bisulfite sequencing. See Figure 2 for the legend on the bar graph for MeDIP. Bottom sub-panel. Single-base DNA methylation levels by bisulfite sequencing. For each sample, 8 randomly-picked clones (rows) were scored for each CpG site (column). Filled circles, methylated CpG sites. Empty circles, unmethylated CpG sites. Upward arrows, cutting sites of the methylation-sensitive restriction enzymes HpaII and HinP1I. (TIFF) [file pone.0015069.s001.tiff]

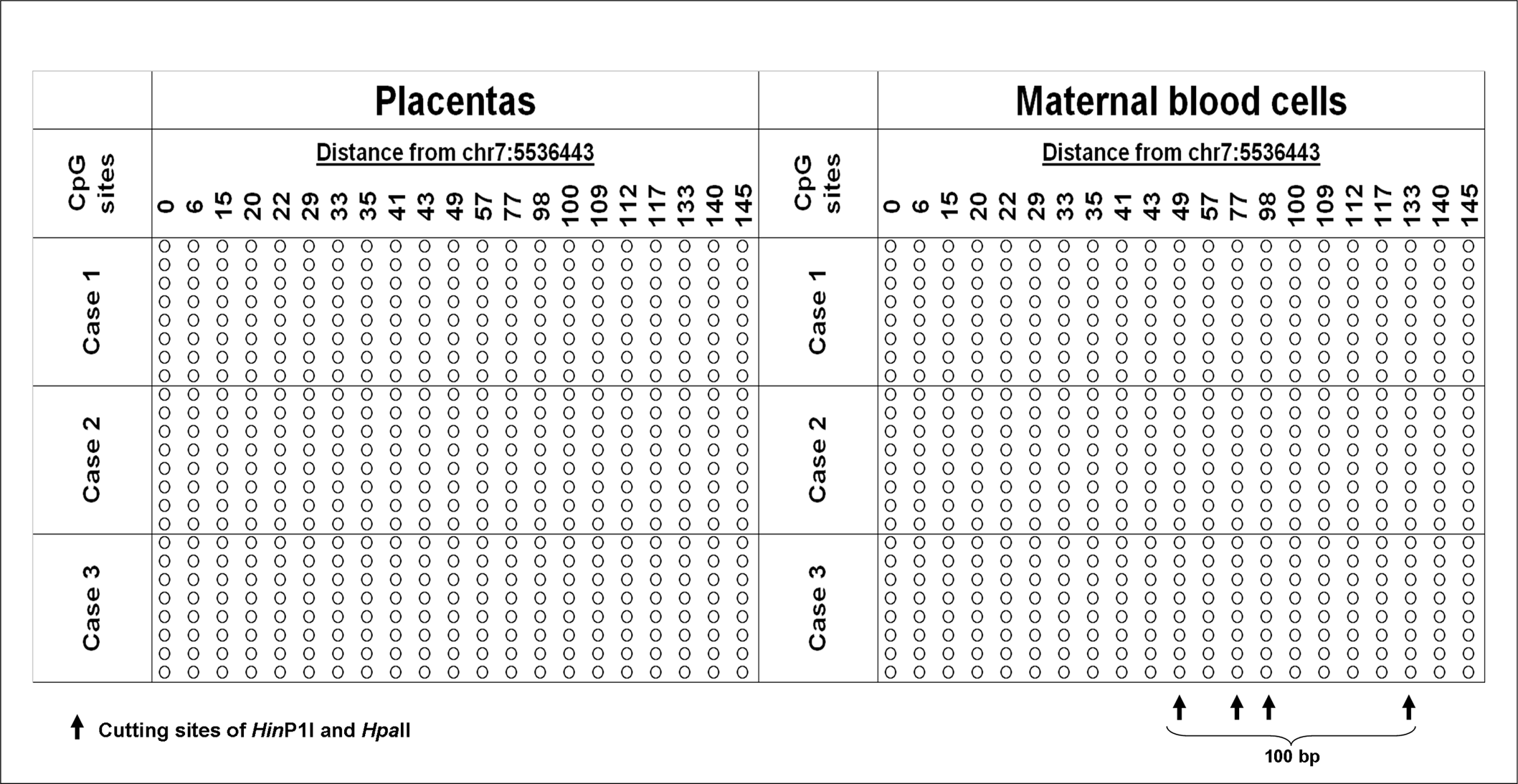

Supplement: Figure S2 — DNA methylation levels by bisulfite sequencing in the β-actin gene. For each sample, 8 randomly-picked clones (rows) were scored for each CpG site (column). Filled circles, methylated CpG sites. Empty circles, unmethylated CpG sites. Upward arrows, cutting sites of the methylation-sensitive restriction enzymes HpaII and HinP1I. (TIFF) [file pone.0015069.s002.tiff]

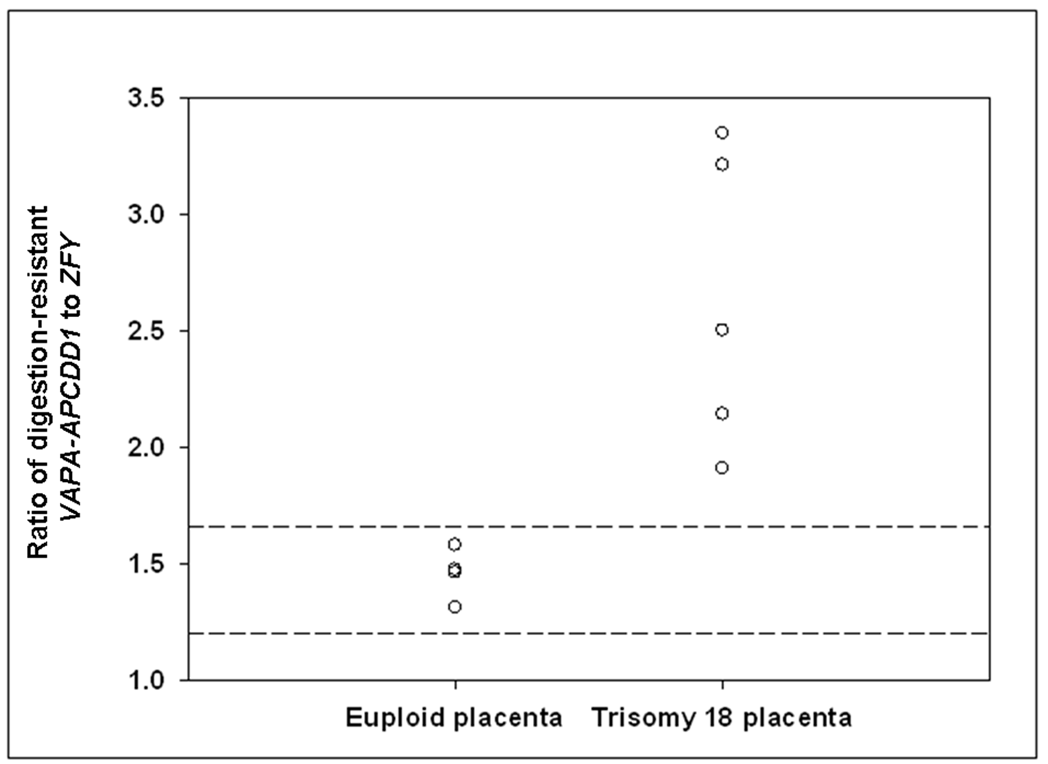

Supplement: Figure S3 — Comparison of chromosome dosage in DNA samples from euploid and trisomy 18 placental tissues. For each sample, the ratio of digestion-resistant VAPA-APCDD1 DNA (chr18) and ZFY DNA (chrY) is plotted. The reference interval of the euploid ratios was calculated as 1.20–1.66 (bound by the dotted lines). (TIFF) [file pone.0015069.s003.tiff]

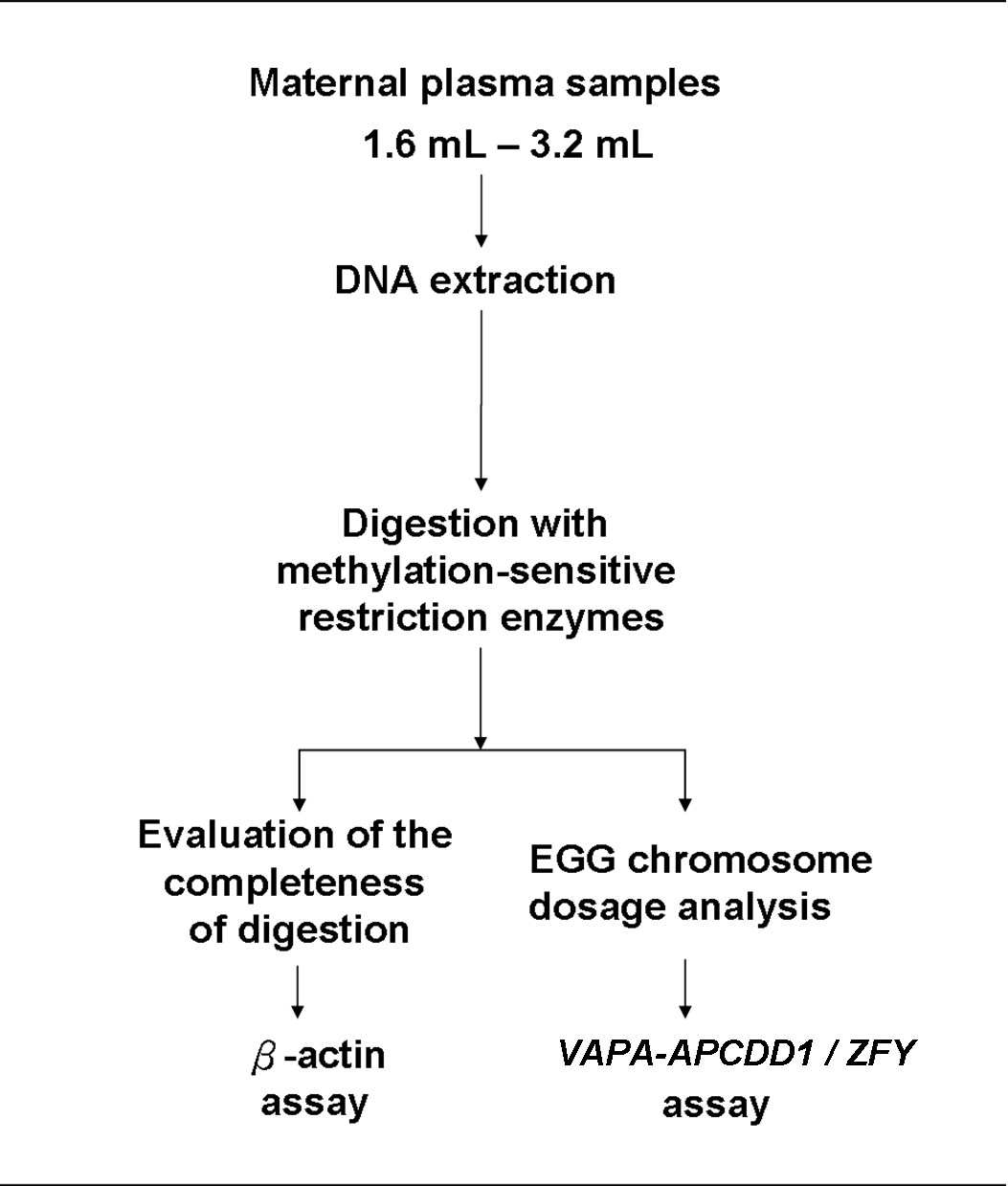

Supplement: Figure S4 — Workflow of the EGG chromosome dosage analysis of maternal plasma samples. Methylation-sensitive restriction enzymes, HinP1I and HpaII. VAPA-APCDD1/ZFY assay, a duplex digital PCR assay. (TIFF) [file pone.0015069.s004.tiff]

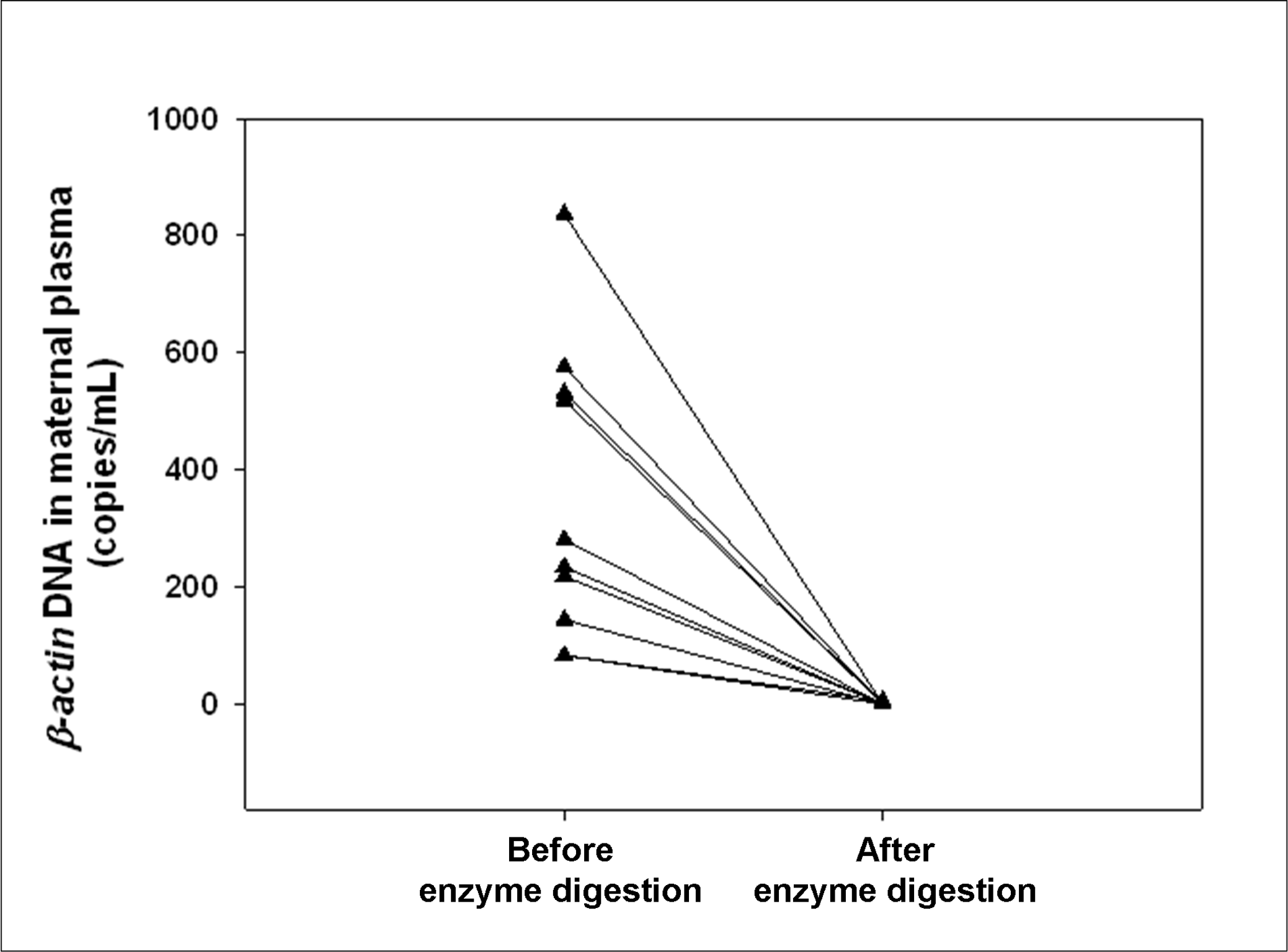

Supplement: Figure S5 — Concentrations of β-actin DNA in EGG-analyzed plasma samples before and after enzyme digestion. β-actin DNA was essentially undetectable by the digital PCR assay in any of the 36 EGG-analyzed plasma samples after digestion. Further analysis of ten maternal plasma samples (8 euploid and 2 trisomy 18 cases) before digestion was also performed by this digital PCR assay. Data of these 10 paired samples are shown. (TIFF) [file pone.0015069.s005.tiff]
