# Supplementary material for: Systematic Identification of Placental Epigenetic Signatures for the Noninvasive Prenatal Detection of Edwards Syndrome
Source: PLoS One. 2010 Nov 30;5(11):e15069. doi: 10.1371/journal.pone.0015069 (PMC2994810; doi:10.1371/journal.pone.0015069)
Supplement: File S1 — Supplementary methods. (DOC) [file pone.0015069.s017.doc]

**File S1**

**SUPPORTING INFORMATION**

**Supplementary Methods**

***Sample recruitment***

Pregnant women attending the Department of Obstetrics and Gynaecology at the Prince of Wales Hospital, Hong Kong, or the Prenatal Diagnostic and Counselling Department at the Tsan Yuk Hospital, Hong Kong, or the Harris Birthright Research Centre for Fetal Medicine, at the King’s College Hospital, London, UK were recruited with informed consent. Plasma from the UK was harvested, kept frozen and sent to Hong Kong in batches on dry ice. Ethical approval was granted by the respective institutional review broads.

***Sample processing***

Maternal peripheral blood samples collected in EDTA-containing tubes were centrifuged at 1,600 × *g* for 10 min at 4°C, and the plasma portion was recentrifuged at 16,000 × *g* for 10 min [1]. The peripheral blood cell portion was recentrifuged at 2,500 × *g*, and any residual plasma was removed. DNA was extracted from maternal peripheral blood cells using the Nucleon Blood DNA extraction kit (GE Healthcare-Biosciences) and from placental tissues using the QIAamp Tissue Kit (Qiagen). DNA was extracted from 1.6 mL of third-trimester maternal plasma or 3.2 mL of first- or second-trimester maternal plasma with the QIAamp DSP DNA Blood Mini Kit (Qiagen) according to the manufacturer’s blood and body fluid protocol and eluted in 50 µL of water.

***MeDIP-chip analysis***

Each DNA sample extracted from placental tissues and maternal blood cells was sonicated and subjected to methylated DNA immunoprecipitation (MeDIP) by antibody directed against 5-methyl-cytosine [2]. The subsequent product was amplified and labeled using the dsDNA terminal labeling kit (Affymetrix) and hybridized to the GeneChip® Human Tiling 2.0R Arrays (Affymetrix). Arrays were washed and scanned according to manufacturer’s standard procedures. Additionally, an “input” control DNA sample without undergoing MeDIP was processed in parallel with the MeDIP-DNA samples. To identify array probes representing the genomic loci with higher DNA methylation in the placenta relative to maternal blood cells, the hybridization signals on each array were analyzed with two data-mining algorithms, namely the Tiling Array Software (TAS) version 1.1 [3] and the Model-based Analysis of Tiling array (MAT) method [4]. We compared the array intensity signals of the placenta relative to those of maternal blood cells.

Using the TAS algorithm, we performed quantile normalization of probe signals by two-sample analysis [3]. An "estimate of fold enrichment" of MeDIP-DNA was calculated as the pseudomedian signal of all MeDIP-sample/“input” control-sample ratios within a 800-bp sliding window. For each sample, the signal for each locus was normalized to give an average value of 1.00 for all loci on the same array. A signal log ratio (SLR) between the median signal of the 5 placentas and that of the 5 maternal blood cell samples was calculated. We expected to obtain a positive SLR for regions that are hypermethylated in the placenta, relative to blood cells. Using a set of parameters in the TAS algorithm (threshold = 1.9, maximum gap = 300 bp and minimum run = 180 bp), we identified loci with a median SLR > 0.4 as hypermethylated in the placenta, compared with maternal blood cells.

Using the MAT algorithm, we performed probe standardization, which predicted the baseline probe behavior to account for the effect of probe sequence context and genome copy number on its signal [4]. The algorithm calculated a P-value to determine if there was a significantly higher array signal (DNA methylation level) in the placenta, compared with maternal blood cells. Using a set of parameters in the MAT algorithm (bandwidth = 400 bp, maximum gap = 300 bp, minimum probe distance = 10 bp), we identified loci with P-value < 10^-5^ as hypermethylated in the placenta, compared with maternal blood cells.

The probes interrogating chromosomes 4, 15, 18 and 20 were tiled on the same array. Thus, only the data from these chromosomes were deposited in the GEO database, because only data from the same array was required for processing and interpreting the data.

***Quantitative DNA methylation analysis by the Epityper***

The assays were performed with a standard MassCLEAVE protocol (Sequenom) [5]. Briefly, 500 ng DNA each from 2 pairs of placental tissues and maternal blood cells was bisulfite-converted using the EZ DNA Methylation Conversion Kit (ZymoResearch). The bisulfite converted DNA was then subjected to PCR amplification by primers listed in Table S1, *in vitro* transcription into RNA, and base-specific RNA cleavage, regardless of the methylation status. Cleavage fragments of different masses would be derived from methylated and unmethylated cytosine, and were resolved and quantified with a MALDI-TOF mass spectrometer (MassARRAY Analyzer Compact, Sequenom).

Epityper assays were designed using the Epidesigner program (version 2.0, Sequenom; [http://www.epidesigner.com](http://www.epidesigner.com/)). Besides picking appropriate bisulfite-PCR primers for a sequence, the program also calculated the masses of the resultant fragments (CpG units), and verified if those masses were within the detection range of the mass spectrometer. The PCR primers were then searched against the bisulfite-converted human genome using the BiSearch search tool [6]. All Epityper assays that might result in more than one PCR products were rejected. The MeDIP-identified loci were considered in terms of the possibility for allowing an appropriate PCR design, and also the number of detectable CpG units in the resultant Epityper assay. We prioritized with the MeDIP-identified loci that could be most effectively analyzed by Epityper assays.

***Cloning and Bisulfite sequencing***

Briefly, 1 µg DNA was bisulfite-converted using the EZ DNA Methylation Conversion Kit (ZymoResearch) and amplified using the same primers used for the Epityper assays (Table S1). They were subsequently TA-cloned into a plasmid vector with the pGEM T-Easy Cloning Kit (Promega). The inserts from the clones were then amplified using vector primers T7 and SP6, followed by sequencing with the BigDye Terminator Cycle Sequencing v1.1 kit (Applied Biosystems) according to manufacturer’s instructions. DNA was then precipitated with ethanol and resuspended in 10 µL of Hi-Di formamide and ran on a 3100 DNA Analyzer (Applied Biosystems). The sequencing data were aligned and scored using the SeqScape software v2.5 (Applied Biosystems). We ensured the conversion rate of non-CpG cytosine residues into uracil residues was >99% in all the regions we tested.

***Digestion of DNA sample by methylation-sensitive restriction enzymes***

The DNA sample extracted from the placenta (50 ng of DNA), maternal blood cell sample (50 ng of DNA) or maternal plasma sample (0.8 mL third-trimester plasma or 1.6 mL first- or second- trimester plasma) was digested by 20 units of *Hin*P1I and 20 units of *Hpa*II in 1X buffer 1 (New England Biolabs) in a total reaction volume of 50 μL. The reaction was incubated for 2 hours at 37°C.

***Conventional qPCR assay for VAPA-APCDD1, ZFY and β-actin DNA***

Quantitative real-time PCR was performed on the 7300 HT sequence detection system based on the TaqMan® hydrolysis probe chemistry (Applied Biosystems). 10 µL of digested DNA were added in a total reaction volume of 50 µL (Applied Biosystems). The primer and probe sequences are listed in Tables S2. Reaction conditions are summarized in Table S3. The limit of detection (LOD) of the assay was tested by subjecting forty replicates of the standard calibrator at a certain LOD concentration. If at least 95% of the wells were positive, that tested LOD concentration could be claimed. Any signals detected below the LOD were considered undetectable.

***Digital PCR assay for VAPA-APCDD1, ZFY and β-actin DNA***

The digital PCR reactions were carried out on the ABI PRISM 7900HT Sequence Detection System. Data were collected by the “Absolute quantification” application in the SDS 2.3.0 software (Applied Biosystems).

***Interpretation of digital PCR data***

We counted the number of wells positive for each target locus. The actual number of molecules distributed into the panel followed the Poisson distribution and was corrected with the following equation: Target = -ln(E/N), wehre Target is the Poisson-corrected counts of the target molecules, E is the number of negative (empty) wells, and N is the total number of digital PCRs in the experiment.

The Poisson equation is given as:


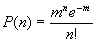


where *n* is the number of template molecules per well, *P*(*n*) is the probability of *n* template molecules in a particular well, and *m* is the average number of template molecules in a particular digital PCR experiment.

The proportion of wells with no template molecule was given by e^-m^. For example, at an average concentration of one template molecular per two wells, the proportion of wells with no template molecule was estimated to be e^-0.5^, which is 60.7% (0.607). The proportion of wells containing one or more template molecules would therefore be 39.4% (100% – 60.7%).

After each digital PCR run, the number of positive wells was counted to calculate the total number of molecules present in the reaction wells according to the following formula:

*No. of molecules = - Ln [(no. of total reactions – no. of positive wells) / no. of total reactions] x no. of total reactions*

The *m* value was calculated by dividing the total number of molecules after Poisson correction by the total number of reactions performed for each run. In the duplex *VAPA-APCDD1 / ZFY* assay, the numbers of positive wells for each fluorescent dye were counted, and the respective *m* value for each marker was calculated separately.

**REFERENCES**

1. Chiu RWK, Poon LLM, Lau TK, Leung TN, Wong EM, et al. (2001) Effects of blood-processing protocols on fetal and total DNA quantification in maternal plasma. Clin Chem 47: 1607-1613.

2. Weber M, Davies JJ, Wittig D, Oakeley EJ, Haase M, et al. (2005) Chromosome-wide and promoter-specific analyses identify sites of differential DNA methylation in normal and transformed human cells. Nat Genet 37: 853-862.

3. Cawley S, Bekiranov S, Ng HH, Kapranov P, Sekinger EA, et al. (2004) Unbiased mapping of transcription factor binding sites along human chromosomes 21 and 22 points to widespread regulation of noncoding RNAs. Cell 116: 499-509.

4. Johnson WE, Li W, Meyer CA, Gottardo R, Carroll JS, et al. (2006) Model-based analysis of tiling-arrays for ChIP-chip. Proc Natl Acad Sci U S A 103: 12457-12462.

5. Ehrich M, Nelson MR, Stanssens P, Zabeau M, Liloglou T, et al. (2005) Quantitative high-throughput analysis of DNA methylation patterns by base-specific cleavage and mass spectrometry. Proc Natl Acad Sci U S A 102: 15785-15790.

6. Tusnady GE, Simon I, Varadi A, Aranyi T (2005) BiSearch: primer-design and search tool for PCR on bisulfite-treated genomes. Nucleic Acids Res 33: e9.
